# The role of auxin during early berry development in grapevine as revealed by transcript profiling from pollination to fruit set

**DOI:** 10.1038/s41438-021-00568-1

**Published:** 2021-06-14

**Authors:** Francisca Godoy, Nathalie Kühn, Mindy Muñoz, Germán Marchandon, Satyanarayana Gouthu, Laurent Deluc, Serge Delrot, Virginie Lauvergeat, Patricio Arce-Johnson

**Affiliations:** 1 Departamento de Genética Molecular y Microbiología, Facultad de Ciencias Biológicas, Pontificia Universidad Católica de Chile, Alameda 340, Santiago, Chile; 2 Facultad de Ciencias Agronómicas y de los Alimentos, Pontificia Universidad Católica de Valparaíso, 2340025 Valparaíso, Chile; 3 Department of Horticulture, Oregon State University, Corvallis, OR 97331 USA; 4 UMR Ecophysiologie et Génomique Fonctionnelle de la Vigne, ISVV, Université de Bordeaux, Villenave d´Ornon, France

**Keywords:** Auxin, Plant development

## Abstract

Auxin is a key phytohormone that modulates fruit formation in many fleshy fruits through the regulation of cell division and expansion. Auxin content rapidly increases after pollination and the manipulation in its levels may lead to the parthenocarpic development. ln *Vitis vinifera* L., little is known about the early fruit development that encompasses from pollination to fruit set. Pollination/fertilization events trigger fruit formation, and auxin treatment mimics their effect in grape berry set. However, the role of auxin in this process at the molecular level is not well understood. To elucidate the participation of auxin in grapevine fruit formation, morphological, reproductive, and molecular events from anthesis to fruit set were described in sequential days after pollination. Exploratory RNA-seq analysis at four time points from anthesis to fruit set revealed that the highest percentage of genes induced/repressed within the hormone-related gene category were auxin-related genes. Transcript profiling showed significant transcript variations in auxin signaling and homeostasis-related genes during the early fruit development. Indole acetic acid and several auxin metabolites were present during this period. Finally, application of an inhibitor of auxin action reduced cell number and the mesocarp diameter, similarly to unpollinated berries, further confirming the key role of auxin during early berry development. This work sheds light into the molecular features of the initial fruit development and highlights the auxin participation during this stage in grapevine.

## Introduction

Fruit formation is a key stage with molecular, cellular, and physiological events that define the final fruit attributes. Studies on the early fruit development show that hormones are key for its regulation^1,2^. However, most studies in grapevine are focused on later stages of the fruit development (*veraison* to maturation), and information about the critical processes occurring during the early grapevine fruit development is scarce. Also, in most studies sampling has not enough resolution within this period. The activation of auxin signaling is an early event occurring soon after pollination/fertilization, which triggers fruit set possibly through the activation of gibberellin biosynthesis, as demonstrated in model species^3–6^. The relevance of auxin has been further corroborated by blocking basipetal auxin transport, which causes indole-3-acetic acid (IAA), the main active auxin, to accumulate in the ovary, thus triggering fruit formation in the absence of pollination^7^. At the molecular level, tomato (*Solanum lycopersicum*) lines silenced for the *Indole Acetic Acid 9* (*IAA9*) gene, which encodes a transcription factor that negatively regulate auxin responses, have precocious fruit set associated with more activated auxin and ethylene signaling^8^. The high percentage of differentially expressed transcripts that are common between pollinated- and auxin-triggered fruit set confirms the connection between early reproductive events and auxin^9^. IAA levels increase as soon as 2 days after pollination (DAP), whereas gibberellin content changes later in tomato fruitlets^10^. In addition, studies have shown a spatiotemporal control of auxin distribution during fruit formation^11,12^. However, little is known about the role of auxin during the early fruit development.

Our understanding of grapevine berry formation and its hormonal regulation is limited, though some key events are known as essential for fruit development, including cell division, cell enlargement, and fertilization^13^. Berry formation begins at anthesis, when flowers are receptive to pollination. Then, pollen tubes grow through the stigma, reach the ovules and fertilize them 2–3 DAP^14^. Stimulation of pollination/fertilization in turn activates cell division and enlargement. Cell division activity peaks the first week after pollination^15,16^. Thereafter, cell division is limited only to peripheral cells, and cell elongation accounts mainly for subsequent increase in fruit size^16,17^.

In grapevine, important hormonal cascades are triggered soon after pollination, involving changes in the transcript abundance of genes associated with phytohormone biosynthesis^18–21^. Gibberellin and auxin have a berry growth promoting effect, and their addition to unpollinated pistils produces parthenocarpic berries^22,23^ or promotes the setting of in vitro grown ovaries^24^. In grapevine, IAA levels are high at anthesis and decrease in coincidence with IAA-aspartate (IAA-Asp) content rise, which is an inactive auxin conjugate^25^. In addition, basipetal auxin transport decreases from fruit set onward^26^, suggesting that auxin homeostasis could be relevant during fruit formation period. As has been mentioned before, the molecular mechanisms connecting pollination/fertilization, auxin-related gene expression, and fruit set are poorly described in grapevine. The present work intends to integrate morphological and reproductive events with transcriptional and auxin-related metabolic data, in order to elucidate the role of auxin during the early fruit development in grapevine, a species whose fruit developmental program may differ from other model fleshy fruits.

## Results

### Characterization of morphological and reproductive events from pollination to fruit set

*Vitis vinifera* cv. red globe emasculated flowers were hand-pollinated at anthesis, when flowers were receptive to pollination (Fig. S1). Carpel/fruit samples were measured or collected at sequential time points that reflected the main processes occurring at early developmental stages, starting from 0 DAP, which was immediately before pollination, to fruit set at 12 DAP.

**Fig. 1:**
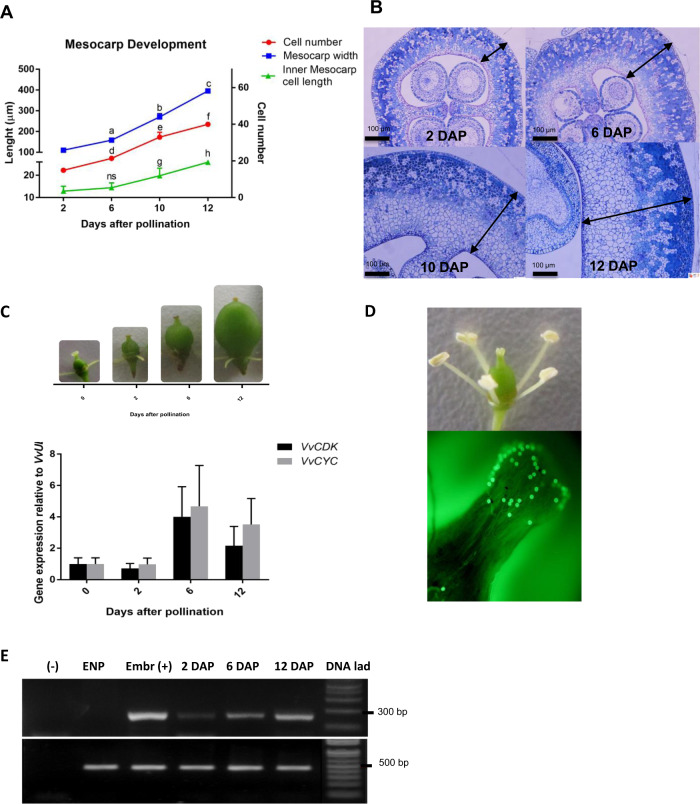
Effect of pollination/fertilization on mesocarp growth in grapevine. **A** Mesocarp width, inner mesocarp cell length, and mesocarp cell number at 2, 6, 10, and 12 DAP. One-way ANOVA was performed and letters indicate statistically significant differences (*p* < 0.05). Bars indicate standard error. ns nonsignificant. **B** Early berry morphology in fruits collected at 2, 6, 10, and 12 DAP. Representative cross-sections observed through light microscopy with toluidine blue stain. Arrow denotes mesocarp width. Scale bars: 100 μm. **C** *VvCYC* and *VvCDK* expression relative to *VvUBI1*. Bars represent standard error from three biological replicates. Representative pictures of the fruits at the different time points are included. **D** Pollen tubes in the stigma and carpel, visualized by callose fluorescence at 520 nm, revealed by aniline blue staining. **E** To monitor the fertilization event, grape embryonic marker Leafy Cotyledon 1 (VvL1L) expression was determined by PCR at 2, 6, and 12 DAP in emasculates non-pollinated flowers (ENP), embryos as positive control, Embr (+), and the negative control (−) (water instead of cDNA for PCR reaction). A 100 pb DNA ladder was used to visualize fragments size (1 kb plus Ladder, Thermo Fisher)

Mesocarp width was measured at 2, 6, 10, and 12 DAP (Fig. 1A), as early fruit growth is due mainly to ovary wall increase, whereas endocarp and exocarp layers, in contrast, contain very few cells at this stage (Fig. 1B). From 2 to 12 DAP, mesocarp width increased by four times, with a higher slope in width curve from 6 DAP to fruit set occurring at 12 DAP (Fig. 1A). Regarding cell length variations, at 10 DAP mesocarp cells were bigger with respect to previous time point, 6 DAP, and cell length continued increasing to 12 DAP reaching more than the double in diameter when compared to 2 DAP (Fig. 1A, B). Cell number steadily increased from 2 DAP onward, with a more pronounced increase from 6 to 10 DAP compared to 2–6 DAP, and then kept increasing from 10 to 12 DAP, although at a slower rate (Fig. 1A). For all parameters shown in Fig. 1A, there is a significant increase at 10 DAP compared to 6 DAP, and 12 DAP compared to 10 DAP. Though not significant, there is a trend toward upregulation of genes coding for a putative cyclin, *VvCYC*, and a putative cyclin-dependent kinase, *VvCDK*, from 2 to 6 DAP (Fig. 1C).

Regarding reproductive events, pollen tubes were present in the style and in the ovary wall at 1 DAP (Fig. 1D). To monitor the fertilization event, grape embryonic marker Leafy Cotyledon 1 (*VvL1L*) expression was determined by PCR. *VvL1L* was expressed from 2 DAP onward, whereas it was not detected in fruits growing from non-pollinated carpels (emasculated non-pollinated, ENP), thus confirming that fertilization took place in the samples obtained (Fig. 1E).

### Global gene expression variations from pollination to fruit set

To generally investigate the main processes taking place in the early fruit development, exploratory RNA-seq analysis^27,28^ was performed on the T0, T2, T6, and T12 samples, where T0 corresponds to unpollinated ovary samples, T2 is 2 DAP sample, T6 is 6 DAP sample, and T12 is 12 DAP sample. Hand-pollination was performed to synchronize berry development. Seventy-two percentage of the reads aligned with the grapevine genome, while 18% presented no match. Approximately 200 million reads were obtained for each time point, and a list of 2822 genes with differential expression (false discovery rate (FDR) < 0.05) and absolute fold change < 2 was generated (Table S2) This list was generated comparing gene expression between all-time points in all possible combinations (T0–T2, T0–T6, T0–T12, T2–T6, T2–T12, and T6–T12).

To better characterize the progressive changes occurring through berry development, a subset of 543 genes with differential expression in sequential time points was generated (FDR < 0.01). Differential expressed genes (DEG) analysis were performed in T0–T2, T2–T6, and T6–T12 comparisons.

**Fig. 2:**
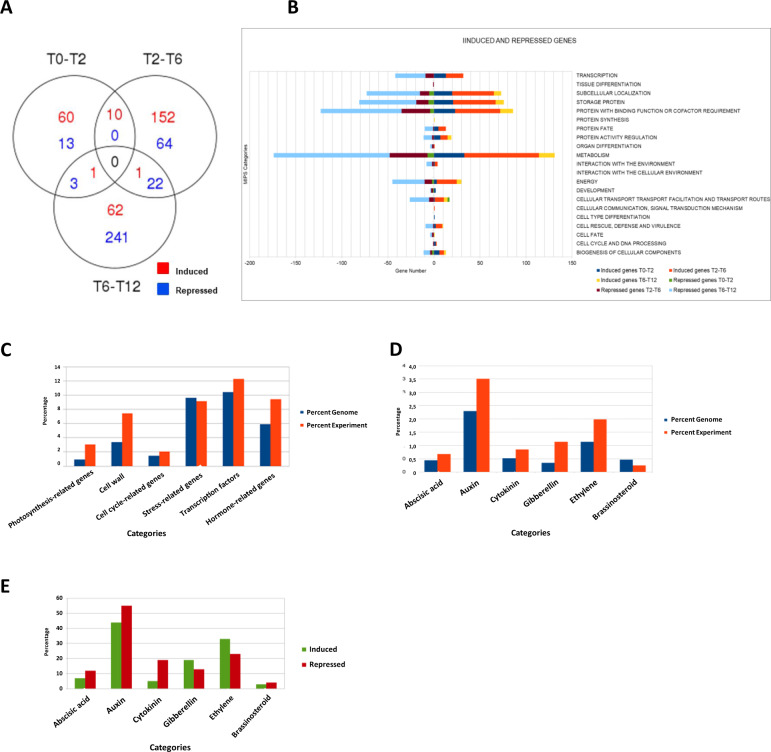
Differentially expressed transcripts during early berry development and overrepresented categories revealed by exploratory transcriptome analysis. **A** Representation of the number of differentially expressed genes at T0–T2, T2–T6, T6–T12 using Venn diagram. **B** Differentially expressed genes at T0–T2, T2–T6, and T6–T12, classified according to MIPS categories. **C** Overrepresentation of selected categories during early stages of berry development found in the genome (blue bars) and in the current experiment (orange bars). **D** Overrepresentation of hormone-specific categories during early berry development in grapevine. RNA-seq analysis was performed on a library generated from one biological replicate formed by a pool of ten carpels/fruits. **E** Overrepresentation of hormone-related categories in early stages of berry development in the genome (blue) and the current experiment (orange)

The number of upregulated genes exceeded the number of repressed genes in T0–T2 and T2–T6, which was reversed in T6–T12 (Fig. 2A). When differentially expressed genes were separated according to MIPS categories, upregulated genes in T2–T6 and downregulated genes in T6–T12 were observed in all categories, whereas upregulation in T0–T2 was exclusive to some categories (Fig. 2B). Analysis of the overrepresented functional categories in the subset of 543 genes showed that the percentage of genes with differential expression reached the percentage of genes that variate in the genome in some categories (Fig. 2C). The three categories with more gene expression changes were transcription factors, stress-related, and hormone-related categories (Fig. 2C). We further analyzed the hormone-related category, which was in turn subdivided in hormone-specific subcategories (Fig. 2D, E). The “auxin” subcategory was overrepresented in the current experiment compared to the other subcategories (Fig. 2D). The most induced and repressed subcategory was “auxin”, followed by “ethylene”, “gibberellin”, and “cytokinin” subcategories (Fig. 2E).

### Auxin-related gene expression changes during early berry development

**Fig. 3:**
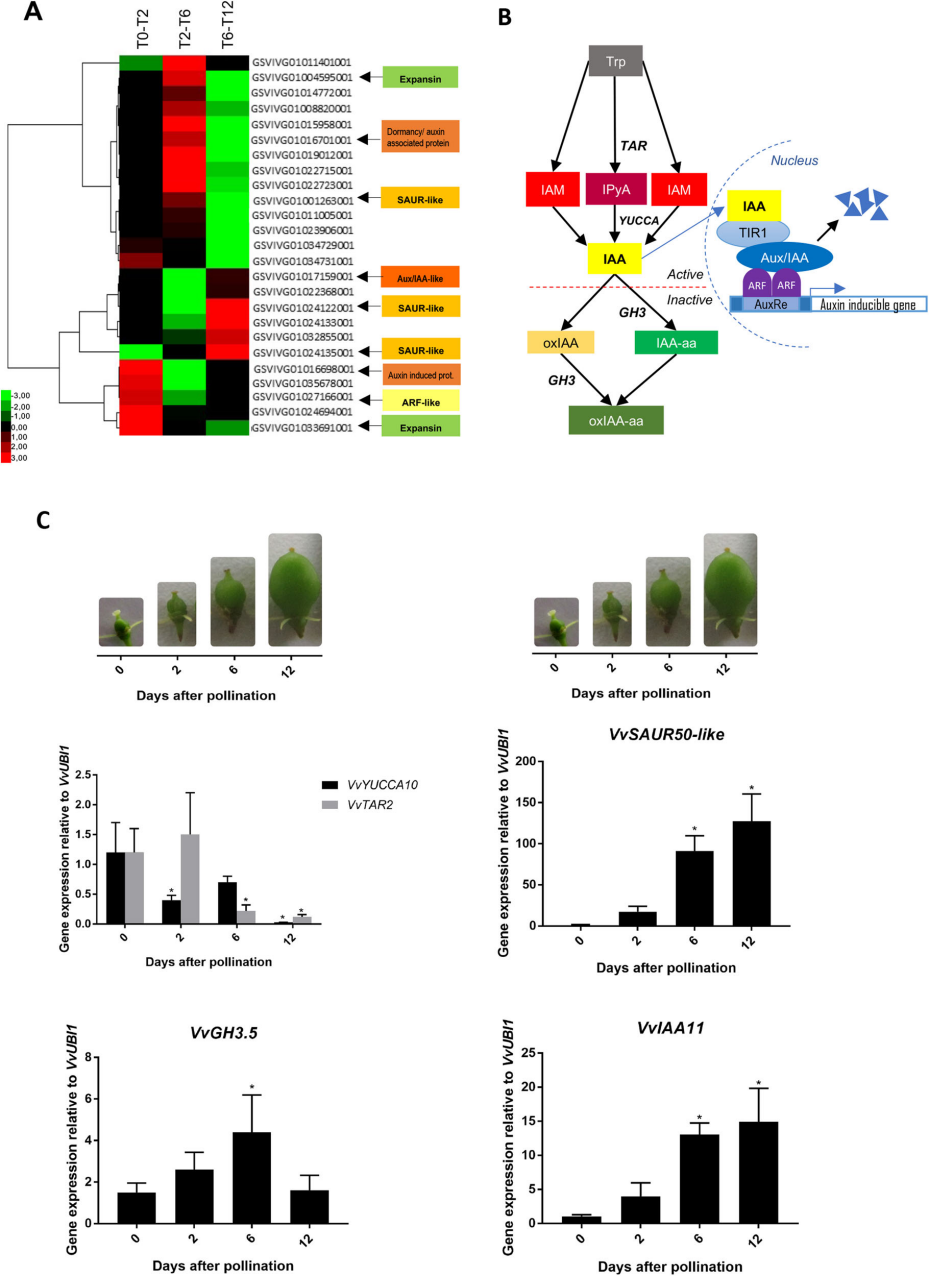
Variations in the expression of auxin-related genes during early steps of berry development. **A** Hierarchical cluster of auxin-related genes during early berry development at T0–T2, T2–T6, and T6–T12 obtained using Cluster 3.0 (Pearson correlation) and TreeView 1.1.6r2. **B** Diagram of the main components of auxin homeostasis and signaling pathways. Colored boxes represent IAA-related compounds, in italic letter the genes associated to the processes indicated by arrows. **C** qPCR analysis of auxin homeostasis-related genes normalized to *VvUBI1*. qPCR analysis of auxin signaling-related genes normalized to *VvUBI1*. In **C**, Student’s *t* test was performed and asterisks indicate statistically significant differences between time points, comparing one time point with the previous one or 0 DAP (*p* < 0.05). Bars represent standard error from three biological replicates. Representative pictures of the fruits at the different time points are included

Considering that auxin was the subcategory most overrepresented within the hormone-related subcategories, and that auxin may have a physiological role, since parthenocarpic development upon auxin treatment has been reported in grape berries^22,23^, we explored the expression profiles of genes within the “auxin” subcategory. As shown in Fig. 3A, several auxin-related genes expressed throughout the initial berry development, with three main gene groups according to their expression patterns: (1) upregulated genes in T0–T2, which then downregulate; (2) upregulated genes only prior to fruit set, in T6–T12, and (3) upregulated genes in T2–T6 which then downregulate in T6–T12 (Fig. 3A).

In order to gain more insight into auxin pathway dynamics, we analyzed the transcript profiles of selected genes possibly related to auxin homeostasis and signaling pathways, depicted in Fig. 3B. Böttcher et al.^29^ reported that members of the TAR and YUCCA families are expressed during grapevine berry ripening, and that increase in TAR gene expression is accompanied by higher IAA and IAA-Asp levels, indicating that this auxin biosynthetic pathway is operative in *V. vinifera*. Putative biosynthetic genes, *VvTAR2* and *VvYUCC10*, possibly involved in the production of IAA (Fig. 3B), decreased their relative expression from anthesis to fruit set, with *VvTAR2* downregulating before (Fig. 3C). In contrast, *VvGH3.5* transcripts, possibly coding for an IAA-amido synthetase^30^ (Fig. 3B), peaked at 6 DAP and then significantly (*p* < 0.05) decreased (Fig. 3C). On the other hand, putative signaling genes from the *Small Auxin Up RNA* (*SAUR*) and *AUXIN/INDOLE ACETIC ACID* (*Aux/IAA*) families—both described as auxin-responsive^31,32^—significantly increased their relative expression from T2 to T6 (*p* < 0.05), which was maintained at T12 (Fig. 3C).

### Auxin metabolites content variations during early berry development

**Fig. 4:**
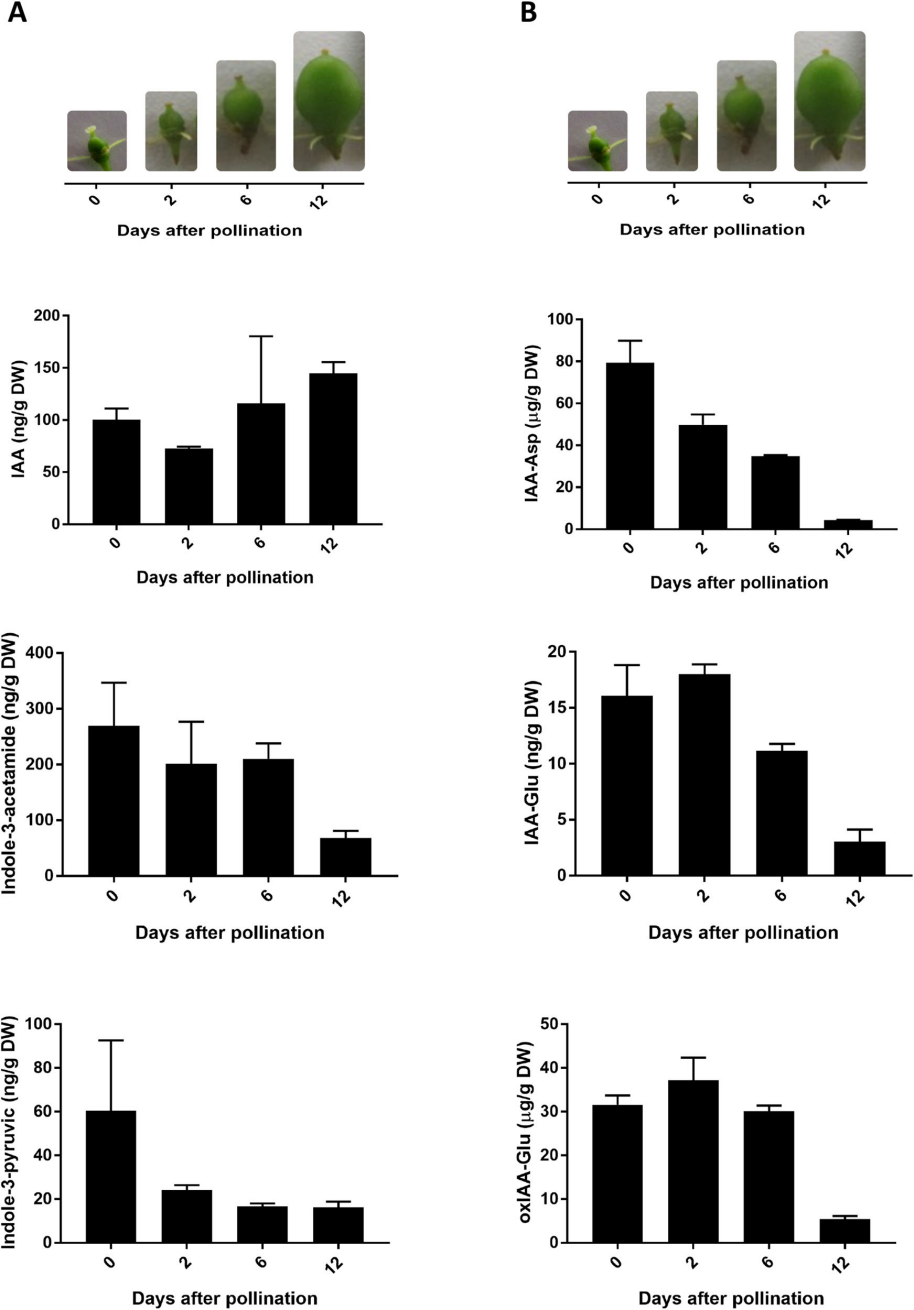
Changes in the auxin and auxin-related compounds content during early grapevine berry development. **A** IAA and IAA precursor levels, and **B** conjugate and oxidized conjugate contents obtained through LC–MS/MS in MRM. Bars represent standard error from two biological replicates. Representative pictures of the fruits at the different time points are included

As auxin-related gene expression presented significant changes during early berry development, we further investigated whether IAA and some of its precursors and conjugates were present during this stage. IAA and the IAA precursors, indole-3-pyruvic acid (IPyA) and indole-3-acetamide (IAM) were detected from 0 to 12 DAP (Fig. 4A).

Free auxin levels are modulated by conjugation with amino acids and other molecules, and by oxidation of IAA and IAA conjugates^33^. Inactive oxidized or conjugated forms allow control of the extent and duration of auxin action, as conjugation is mainly irreversible^34^, so several inactive auxin forms exist. The presence of auxin conjugates during grapevine berry development has been previously reported^25,26^. Aspartate and glutamate conjugates, IAA-Asp and IAA-glutamic (IAA-Glu), were present from 0 to fruit set (Fig. 4B). IAA-Trp was low (<3 ng/ g DW) and IAA-alanine (IAA-Ala) was not detected (not shown). IAA-Asp was the most abundant conjugate (Fig. 4B), while oxindole-3-acetic acid (OxIAA)-Glu content was also very abundant (Fig. 4B).

### Effect of the IAA-Trp treatment, an auxin action inhibitor

**Fig. 5:**
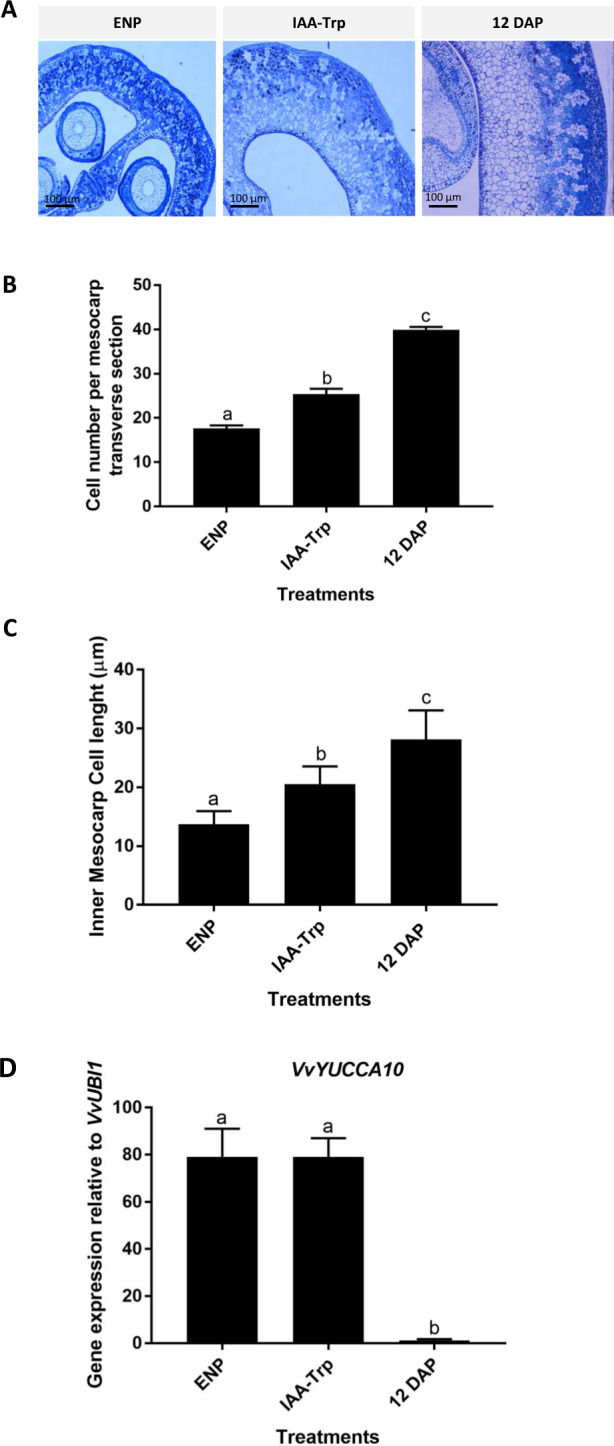
Effect of the inhibition of auxin-related responses using IAA-Trp. **A** Effect of IAA-Trp treatment on mesocarp morphology at 12 DAP. Representative cross-sections of berries grown from emasculated non-pollinated flowers (ENP), pollinated + IAA-Trp (applied at 1 DAP), and pollinated. **B** Effect of IAA-Trp treatment on mesocarp cell number at 12 DAP. **C** Effect of IAA-Trp treatment on mesocarp cell diameter at 12 DAP. **D** Effect of IAA-Trp treatment on the expression of putative auxin biosynthetic gene *VvYUCCA10* relative to *VvUBI1*. One-way ANOVA was performed. Different letters indicate statistically significant differences (*p* < 0.05). Bars represent standard error from three biological replicates

Auxin transcripts exhibit variations during the grapevine berry formation; therefore, we further investigated the role of auxin in this process. For this, we applied IAA-Trp to the fruits. IAA-Trp is an auxin conjugate described as an inhibitor of auxin growth responses^35^. IAA-Trp was applied at 1 DAP, and the effect on mesocarp development was analyzed at 12 DAP. IAA-Trp treatment affected mesocarp size (Fig. 5A). In addition, cell number and length were significantly lower (*p* < 0.05) in IAA-Trp-treated fruits than in 12 DAP untreated fruits, though higher than in fruits developed from emasculated and non-pollinated flowers (ENP; Fig. 5B, C). In order to determine whether the observed effects, i.e., reduced mesocarp size lower cell number and size, were related to changes in auxin biosynthesis-related gene expression, we also measured *VvYUCCA10* transcript abundance at 12 DAP. This gene was significantly (*p* < 0.05) upregulated in ENP and IAA-Trp-treated fruits, compared to 12 DAP untreated fruits (Fig. 5D).

## Discussion

### Berry formation-related morphological and reproductive events are accompanied by distinct gene expression profiles

Pollination and fertilization trigger a complex developmental program that encompasses the transition from a dormant ovary to a developing fruit, which includes the activation of growth through cell division and expansion. We found an increase in the slope of the mesocarp growth curve from 6 DAP, which was paralleled with an augmentation in the rate of cell number increase (Fig. 1A). In addition, cell length changes were first detected at 10 DAP (Fig. 1A). However, from 10 DAP mesocarp growth continued although the increased rate of cell number reduced (Fig. 1A). Based on these results, we hypothesize that before 6 DAP fruit growth relies almost exclusively on cell division, while from 6 DAP onward fruit growth occurs due to both an increase in cell division rate and cell elongation activation. Finally, from 10 DAP berry growth depends mainly on cell elongation, though cell division may contribute, leading to fruit set at 12 DAP. This in in agreement with Ojeda et al.^16^, who measured DNA content of Shiraz berries and found that most of the mitotic activity occurs between the first 15 days after anthesis (DAA). In addition, cell enlargement started 1 week after anthesis, thus within the first week berry growth is almost entirely due to cell division^16^. This differs from other fleshy fruits presenting a sigmoid curve, where cell division occurs during the first 2 weeks and sometimes continues through the whole fruit development. For instance, in tomato, this process occurs after anthesis (approximately during 14 days) and then cell expansion takes place^36^. In apple (*Malus domestica*), the cell division takes place during >4 weeks, and in agreement with this auxin-related genes, such as *ARF*, *GH3*, *Aux/IAA*, and *PIN*, are expressed throughout fruit development^37,38^.

Cell number increase was accompanied with the presence of putative cell cycle-related transcripts *VvCYC* and *VvCDK* from pollination to fruit set, exhibiting a trend toward upregulation (Fig. 1C). On the other hand, the embryonic marker expression, *VvL1L*, was detected at 2 DAP (Fig. 1E). Therefore, possibly, pollination/fertilization may upregulate these genes or other cell cycle-related genes, and subsequently increased cell number from 6 to 10 DAP. In tomato, upregulation of several *CYCs* and *CDKs* was reported from 2 days before anthesis to 4 DAP^9^; thus cell division and cell cycle-related gene expression seem to be very early events during initial fruit development. Cell division activity before 2 DAP (Fig. 1A) may be associated with developmental cues, such as the expression of genes involved in spatial patterning. For instance, *Fleshless berry* mutant (*V. vinifera* L.), with abnormal carpel morphogenesis and a thinner mesocarp, presents differences in the transcript abundance of some developmental genes when compared to WT^39^.

We performed an exploratory RNA-seq for differential global gene expression analysis to reveal a picture of the molecular events occurring in the sequential time points, T0–T2, T2–T6, and T6–T12. Interestingly, more upregulated genes were observed in T0–T2 and T2–T6 than in T6–T12 (Fig. 2A), with a small subset of genes shared (Fig. 2A), suggesting that unique developmental programs activate during the flower-to-fruit transition (T0–T2) and soon after fertilization (T2–T6). By contrast, more downregulated genes were found in the interval T6–T12 than in the previous intervals, some of which were shared with T2–T6. This might suggest that such genetic programs begin to turn off close to fruit set. Remarkably, almost all MIPS categories presented a high number of induced genes in T2–T6 and repressed genes in T6–T12 (Fig. 2B), which suggests that induction followed by deactivation of genes involved in most biological processes is a characteristic feature of fruit formation. In contrast, upregulation in T0–T2 pertains to specific functional categories (Fig. 2B) that are associated with the flower-to-fruit transition, thus it is inferred that more specific processes occur during this stage of development. Possibly there are coordinated developmental programs, influencing cell division and other early processes in T0–T2.

The categories with more percentage of genes that change in the experiment compared to global change were “photosynthesis” and “cell wall synthesis” (Fig. 2C). This is in line with the activation of cellular processes in the mesocarp that are relevant for berry development. For instance, cell expansion requires cell wall modification, and photosynthesis decrease is generally consistent with the transition of the fruit into major sinks, as they approach the ripening phase.

Other categories with more percentage of genes that change in the experiment vs global change “transcription factors” and “hormone-related” categories (Fig. 2C), suggesting the activation of hormone pathways. It is worth noting that many stress responses are hormone-regulated, and transcription factors mediate hormone action in many plant processes, thus the overrepresentation of transcription factors and stress-related categories in our exploratory RNA-seq might be linked with the hormone regulation during berry formation, as previously reported in grapevine^18–20,23^.

The “hormone-related genes” category was subdivided into hormone-specific subcategories with “auxin” category having the highest number of differentially expressed genes, followed by “ethylene”, “gibberellin”, and “cytokinin” (Fig. 2E). In tomato, the most overrepresented categories are “brassinosteroids” and “gibberellin”, and to a lesser degree “cytokinin” and “auxin”, when upregulated genes at 4 DAP are considered^9^. This suggests that grapevine and tomato are different regarding the hormone regulation of fruit set.

Auxin is key for fruit formation since auxin-dependent parthenocarpy growth is to some extent comparable to normal fruit development in several species^6,9,23^. In addition, a hierarchy (chronological/developmental order) in the action of several hormones has been suggested, in which auxin is first activated and then it mediates the expression of gibberellin biosynthesis-related genes^5,9,23^. In tomato, pollination increases auxin content by six times from −1 to 2 DAP^10^. Given that auxin regulates cell division and expansion during fruit formation^36,40^, it may be an important hormone for coordinating molecular and cellular events leading to fruit set.

Integrating the transcriptomic and morphogenic data, T0–T2 possibly includes gene expression variations associated mainly with the flower-to-fruit transition, when cell division is starting to occur, whereas T2–T6 includes gene expression changes produced after pollination/fertilization stimulus, which may lead to the activation of genetic programs that further intensify cell division and activate cell elongation. Finally, in T6–T12 additional molecular events might allow fruit set to occur, while many genes are downregulated, suggesting that some pathways could be turning off. Further investigation should explore these preliminary findings. Exploratory RNA-seq data suggest that the activation of hormonal pathways might be key during berry formation, with a high number of auxin-related genes changing their expression, and auxin playing a prevailing role in the transcript changes during that developmental period.

### Changes in auxin transcripts and metabolites during fruit formation support auxin regulation of cell division and expansion

We further explored the relevance of auxin during fruit formation by identifying the genes within the “auxin” subcategory. We found that members from different families related to auxin signaling and response were expressed during initial fruit development (Fig. 3A), such as *Aux/IAA*, *SAUR*, and *ARF*, suggesting they may be involved in the early fruit development. In grapevine, the *Aux/IAA19* gene has been postulated as a positive plant growth regulator, and presents high transcript levels during anthesis and decreases at fruit set^41^, which is in accordance with the presence of *Aux/IAA* in our exploratory RNA-seq. Also, an *ARF* gene from tomato (*SlARF5*) was reported as a fruit set regulator by modulating auxin signaling pathways^42^. Also, genes encoding for putative expansins (cell wall-loosening proteins related to cell expansion and cell enlargement) were expressed during early berry development (3A), consistent with the mesocarp development observed at fruit set (Fig. 1A, B). This is also in agreement with Dal Santo et al.^43^ who reported expression of several expansin genes during initial fruit development.

Given the results obtained in the exploratory RNA-seq, we analyzed the transcript abundance of selected genes involved in auxin biosynthesis, homeostasis, signaling, and response by quantitative RT-PCR (qPCR; Fig. 3C). *TRYPTOPHAN AMINOTRANSFERASE RELATED* (*TAR*) and *YUCCA* gene families participate in IAA biosynthesis from L-Trp^44–47^ by generating the IPyA intermediate and IAA, respectively (Fig. 3B). The grapevine putative ortholog of a *TAR2* gene was downregulated a few days before fruit set (Fig. 3A). In contrast, *VvYUCCA10* transcript, possibly involved in IAA production (Fig. 3B), was elevated at pollination, at 0 DAP, and then decreased (Fig. 3C), suggesting that auxin biosynthesis could be activated to certain extent before pollination/fertilization. In tomato, the *TAR2* gene and the YUCCA-like *toFZY2* and *toFZY6* genes, encoding YUCCA flavin monooxygenase enzymes, are highly expressed, especially in the embryo, at 4 DPA^11^. This might explain the high levels of auxin at 2 DPA in tomato fruits^10^. Elevated expression of the auxin transporter gene *PIN-FORMED5* (*PIN5*) in tomato embryo cells at 4 DAP^11^, which import IAA into the endoplasmic reticulum, could also explain the high levels of auxin accumulated during the first days of tomato fruit development. This seems to be different from grapevine berries, where the transcript abundance of auxin biosynthesis-related genes decreased during the fruit formation (Fig. 3C), and the reported reduced auxin transport and PIN expression from 7 days after flowering onward in grapevine unseeded fruits^26^. In tomato, two PIN family members are expressed during early berry development with high transcript levels after anthesis, and present lower expression levels at 15 DAA^48^. To further investigate whether these transcript changes were accompanied by auxin-related metabolites variations, we measured IAA and auxin precursors and conjugates metabolites, and found that they were present from pollination to fruit set (Fig. 4A).

Conjugation and oxidation reduce free auxin levels^34^, and a decrease in auxin sequestration or degradation in general associate with IAA content variations. On the one hand, we detected auxin conjugates IAA-Asp and IAA-Glu, and also an oxidized conjugate compound, suggesting that conjugation/degradation might be relevant during early berry development. This is in accordance with what was reported in grapevine early fruit development by Kuhn et al.^26^. It also agrees with the variations in the transcript abundance of the grapevine *Gretchen Hagen.5* (*VvGH3.5*) from 2 to 4 weeks post flowering, characterized as an IAA-amido synthetase for IAA conjugation with amino acids in grapevine berries^30^. In addition, we detected the expression of this ortholog in our samples (Fig. 3C), which increased from 0 to 6 DAP and reduced from 6 to 12 DAP. On the other hand, downregulation of a gene coding for an indole-3-acetate *O*-methyltransferase, an enzyme responsible for the inactivation of auxin through methylation, was recently found to express few DAP in grape berries^19^. Taken together, these observations suggest that sequestration or inactivation mechanisms may be required, in order to maintain auxin homeostasis, and possibly avoid excessive amplification of IAA response after it has been triggered. Along these lines, IAA regulating its own levels by negative feedback loops is well documented^49^.

Although we could only analyze two replicates for auxin metabolite quantification, these results are consistent with those of mesocarp development (Fig. 1A, B), the gene expression findings analyzed by qPCR (Fig. 3C) and with what has been reported by Kuhn et al.^26^. Further analysis should be performed with increased replicate number and more metabolites in order to confirm our results.

**Fig. 6:**
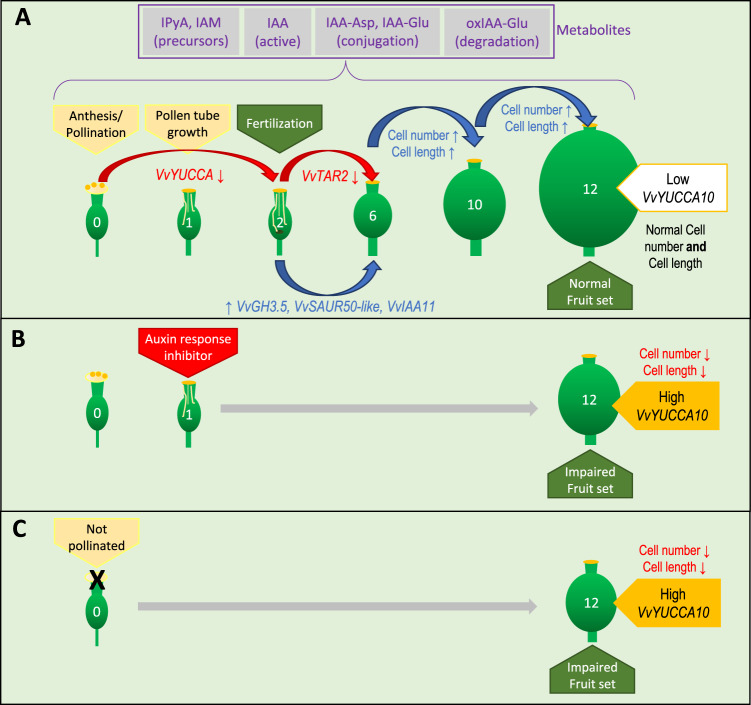
Scheme summarizing the events from pollination to fruit set, and the effect of preventing pollination and the auxin response. **A** Pollination occurred at anthesis, then the pollen tubes were observed at 1 DPA, while fertilization markers were detected from 2 DAP onward. Cell number and length increased significantly from 6 to 10 DAP, and from 10 to 12 DAP, leading to fruit set at 12 DAP. Since exploratory RNA-seq suggested intense gene variation in IAA pathway, we explored the expression of auxin-related genes. From 0 to 2 DAP and from 2 to 6 DAP the expression of the auxin biosynthetic genes *VvYUCCA10* and *TAR2* decreased, respectively, possibly as the auxin response had already been triggered, which is consistent with the increase in the expression of the auxin-responsive genes *VvGH3.5*, *VvSAUR50*, and *VvIAA11* from 2 to 6 DAP. The gene *VvGH3.5* encodes a putative amido synthetase, which could also contribute to the control of the IAA levels after the auxin response activation. Dynamic metabolism of auxin is revealed by the presence of IPyA and IAM precursors, and the conjugates IAA-Asp, IAA-Glu, and OxIAA-Glu from pollination to fruit set. **B** When the auxin response inhibitor IAA-Trp was applied at 1 DAP, cell number and length decreased at 12 DAP compared with control fruits, and the expression of *VvYUCCA10* was higher, which suggests that the auxin response inhibition might be counteracted with more auxin production. **C** A similar effect occurred when pistils remained unpollinated, where fruit set was even more impaired. Integrating **A**, **B**, and **C**, we propose that most of the fruit sizing effect of pollination is mediated by auxin during the grapevine fruit formation. Numbers within the fruits indicate the time after pollination (0, 1, 2, 6, 10, and 12 days after pollination, DAP)

Orthologs of auxin-responsive genes from *SAUR, GH3*, and *Aux/IAA* families, including *VvSAUR50-like*, *VvIAA11*, and *VvGH3.5*, increased their expression significantly (*p* < 0.05) from 2 to 6 DAP, and *VvSAUR50-like* and *VvIAA11* maintained high expression at 12 DAP (Fig. 3C), strengthening the implication of auxin within this period. Interestingly, a gene encoding a putative negative regulator of auxin pathway belonging to *Aux/IAA* family of transcription factors, *VvIAA11*, was upregulated from 2 to 6 DAP (Fig. 3C). As Aux/IAA proteins antagonize auxin responses, this upregulation might represent a negative feedback for slowing the auxin response. In agreement with our results, in tomato several auxin-responsive signaling-related genes upregulate around fruit set^9^. While auxin biosynthesis seems to be inhibited from pollination to fruit set through downregulation of *VvTAR2* and *VvYUCCA10*, increased transcripts of *VvGH3.5* could indicate that IAA levels are controlled by different mechanisms after fruit set. Taking together, these results suggest that a balance between deactivation and induction of genetic programs fine tunes berry set in the grapevine. Figure 6 summarizes the main findings of this work at the physiological and molecular levels.

The treatment with IAA-Trp, an inhibitor of auxin action^35^ reduced mesocarp thickness and cell number and diameter, resembling to some extent the lack of pollination of fruits developed from ENP flowers (Fig. 5A, B). Interestingly, the relative expression of *VvYUCCA10* was significantly higher in IAA-Trp-treated fruits (*p* < 0.05) compared to untreated fruits. Possibly, an affected auxin response might lead to the activation of auxin biosynthesis, in order to maintain normal fruit developmental programs.

Based on our results, the molecular events mediating grapevine berry fruit set could be temporally and morphologically distinct from other fleshy fruit models. First, we suggest that pollination/fertilization takes fewer days to activate cell division and elongation in grapevine berries than tomato and apple. This effect may be related to the upregulation of auxin response-related genes after pollination/fertilization. On the other hand, some changes occurring soon after anthesis seems to be related to developmental cues, in which auxin may be also relevant.

Auxin was the most overrepresented category in the exploratory transcriptomic analysis, therefore in grapevine fruit set auxin seems to be more relevant than in other fleshy fruits, and this is consistent with the poor mesocarp development observed when IAA-Trp was applied (as indicated by decreased cell number and length, Fig. 5B, C). On the other hand, between 2 and 6 DAP, auxin-responsive gene expression (*VvGH3.5*, *VvSAUR50-like*, and *VvIAA11*) was high in grapevine berries (Fig. 6). Auxin biosynthesis might be repressed soon after pollination/fertilization to maintain adequate IAA levels, and then other mechanisms, such as conjugation or transport could maintain IAA levels necessary to activate genetic programs, leading to fruit set. Inhibition of auxin responses affects mesocarp growth in grapevine, as shown by the IAA-Trp treatment, where it caused a reduction in cell division and expansion. However, further research is needed in order to confirm and gain more insight into the key role of auxin during early berry development.

## Materials and methods

### Plant material and controlled pollination

*V. vinifera* L. var. red globe plants were randomly selected from an experimental field in Curacaví Valley, Chile (33° 36′ S, 70° 39′ W) during the 2011/2012 and 2012/2013 growing seasons. Ten bunches per tree, with ~200 flowers each, were used for controlled pollination, where each tree is a biological replicate. Bunches and trees similar in their developmental status were selected. Flowers were carefully emasculated using tweezers at 7–10 days before anthesis, which was recognized as the time when the stigma releases a sticky solution^50^. At anthesis flowers were hand-pollinated with pollen from the same variety and subsequently covered with a paper bag to avoid pollination by opened flowers nearby. Details of the procedure are shown in Fig. S1. Ovaries and fruits samples were collected at T0, T2, T6, and T2, which is 0, 2, 6, and 12 DAP, respectively. T0 corresponds to anthesis, immediately before pollination, T2 is early post-pollination/fertilization, T6 is post-pollination/fertilization, and T12 corresponds to fruit set. Samples were immediately frozen in liquid nitrogen and stored at −80 °C until RNA isolation or extraction for auxin metabolite determinations. All samples were collected at the same time of the day.

### Sample fixation and microscopy

For pollen grains and pollen tube visualization in the stigma, 1 DAP carpels were fixed in FAA (5% glacial acetic acid, 3.7% formaldehyde, and 50% ethanol). Callose in the pollen was revealed by aniline blue staining, where callose fluorescence was detected at 520 nm, according to Longbottom et al.^51^, in a Nikon microscope Eclipse 80i (Nikon, Japan) using NIS Element software.

For carpel/berry mesocarp growth observations, samples collected at 2, 6, 10, and 12 DAP were fixed in FAA solution. Afterward, fixed samples were vacuum treated and passed through increasing ethanol series for tissue dehydration, then paraffin-embedded and cut into 6–8 μm transverse sections in an HM 325 Rotary Microtome (Thermo Scientific™, Thermo Fisher Scientific, USA). Sections were stained with 0.05% toluidine blue in sodium acetate buffer. Images were obtained using a light microscope (Olympus BX51). Cell number per transverse section, cell diameter, and mesocarp width were obtained through tissue observation using the open-source software ImageJ®^52^. For mesocarp width, cell number, and diameter, a row of cells from inner epidermis to outer epidermis was measured (including inner plus outer mesocarp). These parameters were determined in eight biological and two technical replicates per time point. Technical replicates were averaged.

### RNA extraction, cDNA synthesis, and PCR analysis

RNA extraction was performed as described by Poupin et al.^53^. For total RNA extraction from 200 mg of frozen tissue, the CTAB-spermidine method was used. DNase treatments were achieved using Ambion®, TURBO DNA-free™ DNase (Invitrogen^TM^, Thermo Fisher Scientific, USA), following the manufacturer’s instructions.

For cDNA synthesis, 1.5 µg of total RNA was obtained using SuperScript™ II reverse transcriptase (Invitrogen^TM^, Thermo Fisher Scientific, USA), according to the manufacturer’s instructions: 1.5 µg of previously DNase-treated RNA were mixed with 50 ng of random hexamers and 1 µL of dNTP mix (10 mM) in a final volume of 12 µL. Samples were incubated at 65 °C for 5 min, and then transferred immediately to ice, before addition of 4 µL of 5× first-strand buffer and 2 µL of 0.1 mM DTT (Invitrogen^TM^, Thermo Fisher Scientific, USA). After incubation, 2 min at 25 °C, 1 µL of SuperScript™ II reverse transcriptase (Invitrogen^TM^, Thermo Fisher Scientific, USA) was added, and samples were incubated for another 10 min at 25 °C, 50 min at 42 °C, and finally 15 min at 70 °C. RNA integrity was assessed through electrophoresis in denaturing conditions, using MOPS buffer. RNA quality was spectrophotometrically assessed, with A260/A280 and A260/A230 ratio between 2.0 and 2.1.

PCR reactions were made in a final volume of 20 µL with Taq DNA polymerase (Invitrogen^TM^, Thermo Fisher Scientific, USA). Buffers and primer concentrations were as recommended by the supplier. After a denaturation step at 94 °C for 3 min, 35 cycles of the following steps were performed: 30 s at 94 °C, 30 s at 55–57 °C, and 60 s per 1000 nucleotides at 72 °C, and a final elongation step at 72 °C for 10 min. Samples were analyzed by 1% agarose gel electrophoresis using TAE buffer^53^.

### Quantitative comparison of gene expression

qPCR analysis of genes coding for putative cell cycle proteins and components of auxin homeostasis and signaling pathways was performed. Specific primers (Table S1) for amplifying 80–120 pb fragments of these gene were designed using Primer3plus^54^. qPCR was executed as described by Poupin et al.^53^, using a Stratagen Mx3000P instrument and SensiMix™ Plus SYBR® commercial kit (Quantace, London, UK), according to the manufacturer’s instructions.

*VvUBIQUITIN1* (*VvUBI1*, TC53702, TIGR database, VvGi5) and *VvACTIN* (NCBI database XM_002282480.2) gene were used for qPCR normalization^55^. *VvUBI1* presented Ct inter-samples variations <1.5, so it was selected for representing the relative transcript abundance in the graphs. Relative gene expression calculations were conducted using the comparative Ct method^56^, following the MxPro QPCR Software (Agilent, USA) manufacturer’s instructions, where an accurate ratio between the expression of the gene of interest and the gene selected for normalization (*VvUBI1*) was performed. To allow an easier comparison between samples, the expression of the T0 sample was arbitrarily set to 1.0. All experiments were carried out using three biological and two technical replicates, where a biological replicate is a pool of ten ovaries/berries from a berry cluster. Details regarding the primers are provided in Table S1 (Supplementary Material).

To monitor the fertilization event, grape embryonic marker Leafy Cotyledon 1 (VvL1L) expression was determined by conventional PCR analysis, using primers reported previously^57^. Fruits samples at different DAP were compared with fruit samples from ENP flowers at 12 DAP and with the embryo at torpedo stage. Fruit and embryo RNA extraction and cDNA synthesis was performed as described.

### Exploratory RNA-seq analysis

For the exploratory RNA-seq one biological replicate, consisting of a pool of ten carpels/berries (depending on the time point), was collected to produce the RNA-seq libraries of T0, T2, T6, and T12 time points. RNA of whole carpel or fruit (including fertilized ovules) with RIN (RNA integrity number) > 7.0 was used for cDNA library construction, and the expression profiles obtained were mesocarp enriched, though the expression of other tissues might be present as traces, including exocarp, endocarp, epidermis, and embryo. Library construction and sequencing was performed by Macrogen Inc. (South Korea) using the Illumina HiSeq 2000 platform, and 100 bp paired-end reads were generated. The obtained reads were mapped to the grapevine 12× reference genome (ftp://ftp.jgi-psf.org/pub/compgen/phytozome/v9.0/Vvinifera/annotation/), using TopHat 2.0.4 (ref. ^58^). Reads that presented multiple matches or were misaligned were discarded.

For exploratory differential gene expression analysis between time points (0, 2, 6, and 12 DAP) Cufflinks/Cuffdiff 2.0.2 (FDR 0.05) was used^59,60^ (Fig. S4), obtaining significant FPKM values. Genes that presented an absolute fold-change value of at least two between different time points with an adjusted *p* value of 95% significance or higher were selected as DEG. A list of 2822 genes was generated, from which a sublist of 543 genes had differential gene expression between sequential time points (T0–T2, T2–T6, and T6–T12). These genes were used for subsequent analysis.

Gene Ontology (GO) categories were assigned by Blast2GO^61^ with a FDR cutoff of 0.05% probability to the complete list (2822 genes). MIPS categories were assigned to the obtained sublist of sequential time points, composed of 543 genes (http://www.helmholtz-muenchen.de/en/ibis).

Cluster 3.0 was used with Pearson correlation for cluster creations (Cluster 3.0). MeV 4.8.1 (MultiExperiment Viewer)^62^ was used to generate expression profiles (with the *k*-means method), and Treeview 1.1.6r2 for visualization.

### Auxin metabolites determination

Two biological replicates, consisting in a pool of ten ovaries or berries (depending on the time point) were collected as described in subsection 2.1. Hormone extraction was performed according to methods describe by Gouthu et al.^63^. Briefly, 50 mg of lyophilized tissue were extracted in 3 mL of extraction solvent (methanol: formic acid: water, 15:1:4), and 100 μL of internal standard solution containing 20 ng of each standard was added. Whole carpel/fruit was extracted, including ovules/embryo.

Hormone analysis was performed on a hybrid triple quadrupole/linear ion trap 4000 QTRAP LC–MS/MS instrument equipped with a Turbo V source (Applied Biosystems, USA). The analytical method used was LC–tandem mass spectrometry in multiple reaction monitoring mode (LC–MS/MS in MRM) by comparison with standard curves. Retention time and transition are included in Table S2 (Supplementary Material).

Standards for IAA, IAA-Asp, IAA-Ala, and IAA-Glu were purchased from OlChemIm Ltd., (Olomouc, Czech Republic). IPyA and IAM were purchased from Sigma. Standards for OxIAA and OxIAA-Glu were kindly provided by Dr. Hisashi Miyagawa (Division of Applied Life Sciences, Graduate School of Agriculture, Kyoto University, Japan). The following internal standards purchased from OlChemIm Ltd. were used for each compound, as described in Gouthu et al.^63^: for IAA, IPyA, and IAM, (^2^H_5_)IAA; for IAA-Glu, (^2^H_5_)IAA-(^15^N)Glu; for IAA-Asp and IAA-Ala, (^2^H_5_)IAA-(^15^N)Asp; for IAA-Trp, (^2^H_5_)IAA-(^15^N)Tr; for OxIAA-Glu, (^2^H_2_)OxIAA-Glu was used; and for OxIAA, (^2^H_2_)OxIAA, provided by Dr. Miyagawa.

### IAA-Trp treatment

A total of 1 µM IAA-Trp (OlChemIm Ltd., Olomouc, Czech Republic), used as an auxin action inhibitor as reported^35^, was applied at 1 DAP. A mixture of lanoline:baseline (1:1) was used to apply the IAA-Trp treatment. IAA-Trp treated fruits were compared with fruits from pollinated flowers and fruits from ENP flowers at 12 DAP. Three bunches from three different plants were treated. For sampling at 12 DAP, a pool of selected fruits from the three bunches was made, where each plant is a biological replicate.

## Conclusion

This work gives a broad picture of the molecular events related to the auxin dynamics during grapevine berry formation, where some differences may be appreciated with model species for fleshy fruit formation. Auxin is crucial for berry formation as shown by the extensive auxin-related gene expression changes, which could explain the activation of cell division and expansion, among other processes. In fact, qPCR analysis revealed that auxin homeostasis and response-related genes changed, and several auxin metabolites were present, from pollination to fruit set. Finally, the effect of the inhibitor of auxin response, IAA-Trp, on fruit growth further confirmed the importance of auxin during this stage of development. This knowledge will help understand auxin participation in early grape berry formation, and could set the basis for the development of new agronomic practices to improve grapevine berry traits.

## Supplementary information

Supplementary Material
