# Supplementary material for: The role of auxin during early berry development in grapevine as revealed by transcript profiling from pollination to fruit set
Source: Hortic Res. 2021 Jun 14;8:140. doi: 10.1038/s41438-021-00568-1 (PMC8203632; doi:10.1038/s41438-021-00568-1)
Supplement: Supplementary file 1 — Supplementary Material [file 41438_2021_568_MOESM1_ESM.docx]

The role of auxin during early berry development in grapevine as revealed by transcript profiling from pollination to fruit set

Francisca Godoy^1^, Nathalie Kühn^2^, Mindy Muñoz^1^, Germán Marchandon^1^, Satyanarayana Gouthu^3^, Laurent Deluc^3^, Virginie Lauvergeat^4^, Serge Delrot^4^, Patricio Arce-Johnson^1 *^

1. Departamento de Genética Molecular y Microbiología, Facultad de Ciencias Biológicas, Pontificia Universidad Católica de Chile, Alameda 340, Santiago, Chile.
2. Facultad de Ciencias Agronómicas y de los Alimentos, Pontificia Universidad Católica de Valparaíso, 2340025 Valparaíso, Chile.
3. Department of Horticulture, Oregon State University, Corvallis, OR 97331, USA.
4. UMR Ecophysiologie et Génomique Fonctionnelle de la Vigne, ISVV , Université de Bordeaux, Villenave d´Ornon, France.

*: Corresponding author, parce@bio.puc.cl

1. **Supplementary material**


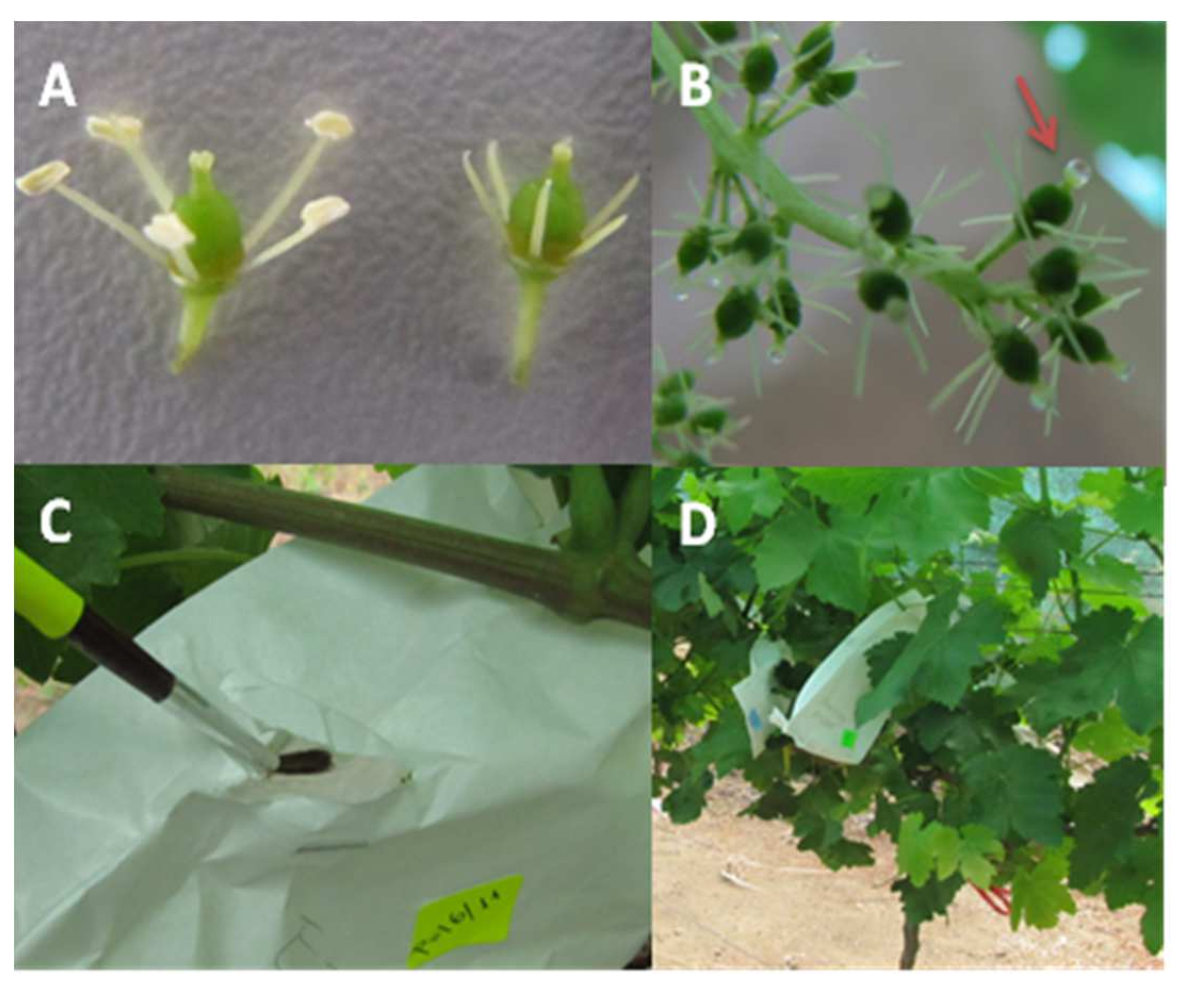


**Fig. S1: Emasculation and hand-pollination technique on *Vitis vinifera* cv. Red Globe flowers**

(A) Flowers of *Vitis vinifera* cv. Red Globe were emasculated. Left: open flower with its anthers (non-emasculated). Right: flower after anthers removal. (B) Approximately five to seven days after emasculation, flowers were receptive to pollination (drop on the stigma, red arrow) and covered with a paper bag to avoid pollination from nearby flowers. (C) Receptive flowers were hand-pollinated and (D) samples at different time points were collected.


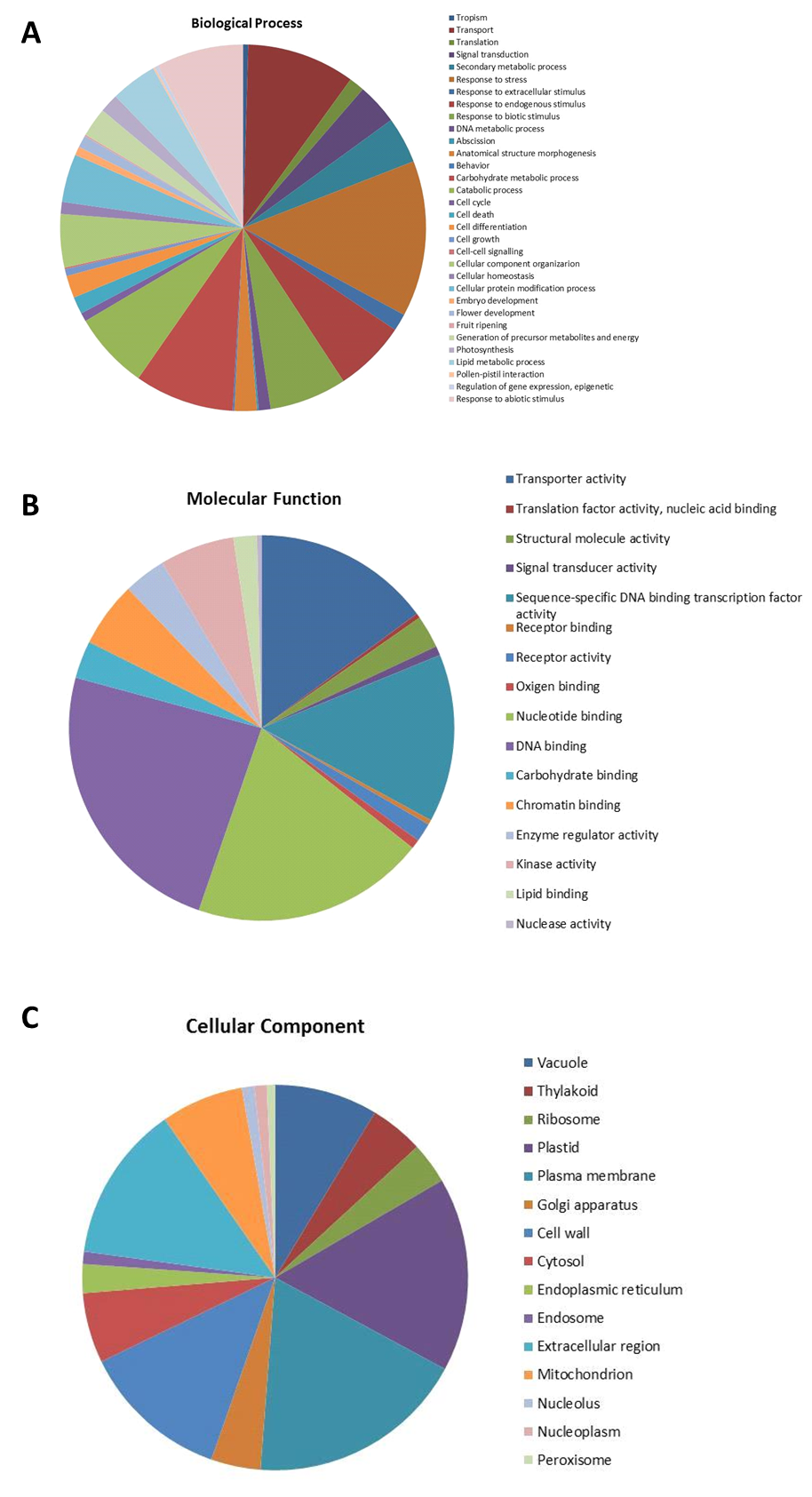


**Fig. S2:** **Gene Ontology distribution of differentially expressed genes found during sequential time-points of initial berry development.**

GO annotations for differentially expressed genes during initial berry development in grapevine were assigned using Blast2GO. (A): Biological Process. (B) Molecular Function. (C) Cellular Component.


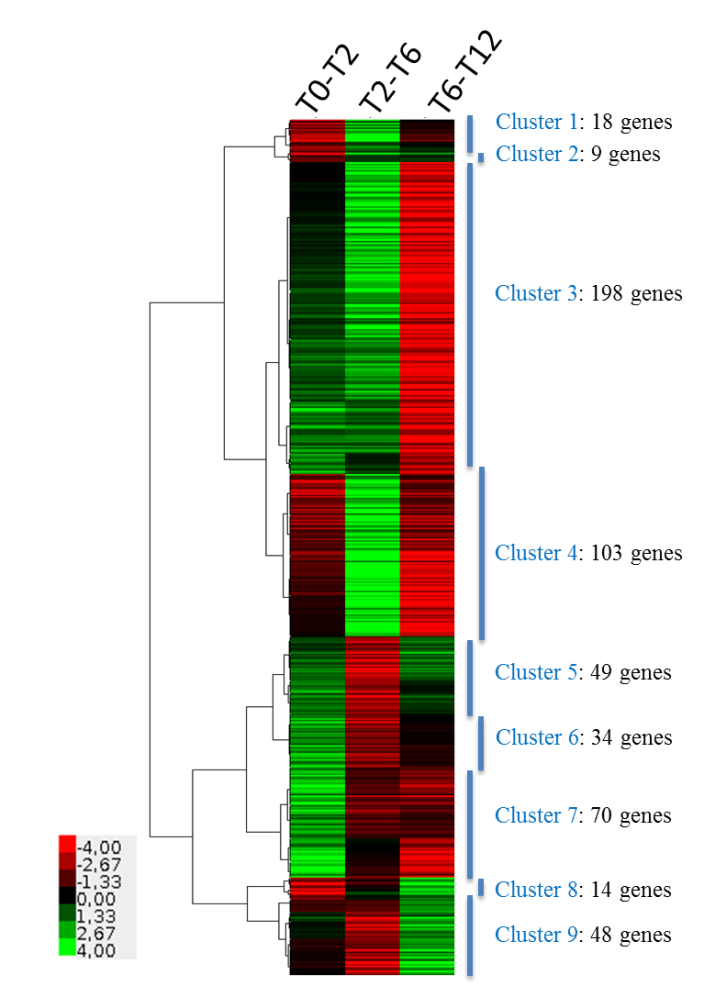


**Fig. S3**: **Cluster of differentially expressed genes throughout early berry development**

Overview of the hierarchical cluster display of differentially expressed genes for sequential time-points during early berry development was obtained using Cluster 3.0 (Pearson correlation) and TreeView 1.1.6r2. Nine distinct expression patterns are distinguished.


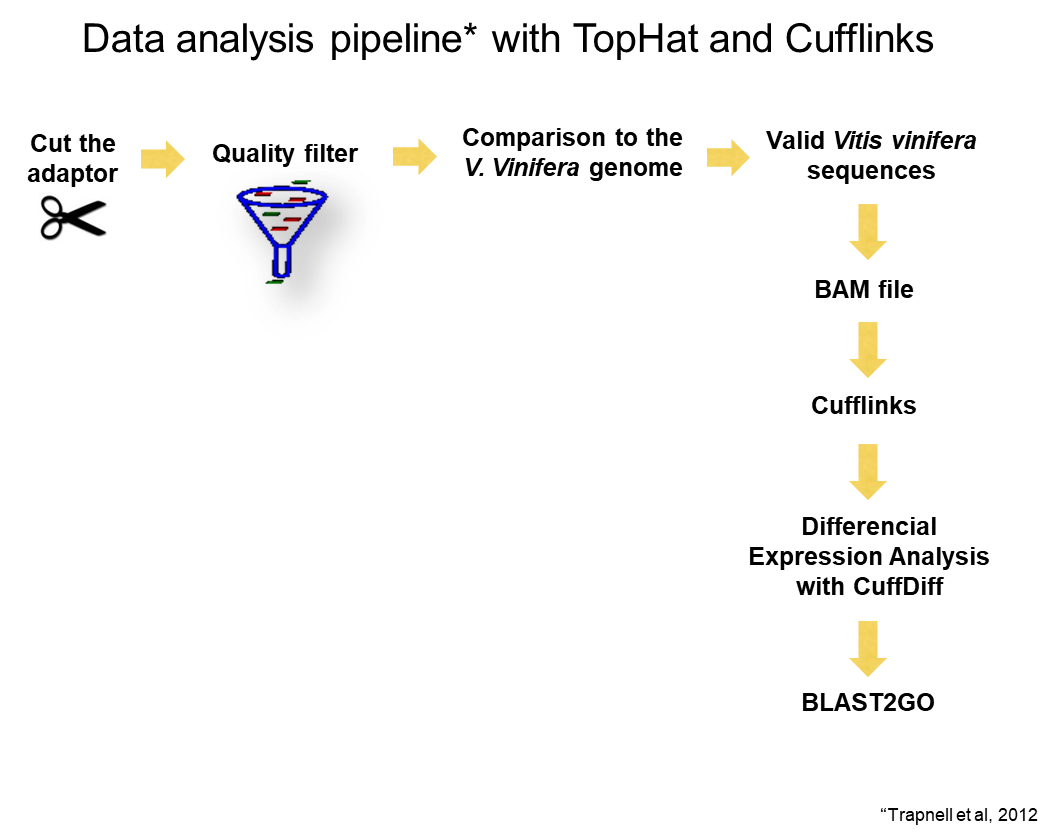


**Figure S4: Exploratory RNAseq pipeline**

Overview of the methodology used for the exploratory RNAseq experiment, based on Trapnell et al (2012).

**Table S1: Primers used for gene amplification.**

| **Primer Name** | **Sequence** | **Tm (°C)** | **Grapevine ID** | **Size product (bp)** |
| --- | --- | --- | --- | --- |
| **qSAUR50-like-F** | CAGTCCTCAAGAAGTGGCCC | 58 | GSVIVG01016698001 | 249 |
| **qSAUR50-like-R** | GCAAGTGCTCGAACAGAACC | 57 |  |  |
| **qVvGH3.5-F** | TGCTGATGCCTGTGATGAAC | 55 | GSVIVT01000422001 | 146 |
| **qVvGH3.5-R** | TTTGACACAGCCCACAAAGC | 56 |  |  |
| **qVvYUCCA10F** | CAGGGGAAAATGGAATGTTGGG | 56 | GSVIVT01011005001 | 94 |
| **qVvYUCCA10R** | ACAAACGCATCGCTCGTTTC | 56 |  |  |
| **qIAA11-F** | TTCTATTGGCTGCCGGTTGT | 57 | GSVIVG01027166001 | 226 |
| **qIAA11-R** | AGCACGACCAACTGCTACTC | 57 |  |  |
| **qTAR2F** | GGGGGACATAACAACCATTG | 54 | GSVIVG01007679001 | 74 |
| **qTAR2R** | GGCTCCAAAAACCAGCATAG | 54 |  |  |
| **qVvActin-F** | TCCTTGCCTTGCGTCATCTAT | 55 | XM_002282480.2 | 80 |
| **qVvActin-R** | CACCAATCACTCTCCTGCTACAA | 56 |  |  |
| **qVvCDK-F** | GCAAGGTTTACAAGGCCAAG | 57 | XM_002266587.1 | 81 |
| **qVvCDK-R** | CTTCCTCGTCCATTTCAAGG | 57 |  |  |
| **qVvCYC-F** | AGCCAAAGCCTGAACAAGTG | 56 | XM_002283116.1 | 85 |
| **qVvCYC-R** | CTGCCCTTTTTCTTGCTCAC | 56 |  |  |

Table S2: Retention time and transitions of auxin metabolites

Positive mode

| Metabolite | RT | RT (IS) | Transition | Transition (IS) |
| --- | --- | --- | --- | --- |
| IAM | 12,5 | no | 175 > 130 | no |
| IPyA | 12,76 | no | 204 > 136 | no |
| IAA | 17 | 17,83 | 176 > 130 | d5: 181 > 134 |
| IAA-Asp | 14 | 13,87 | 291 > 130 | d5: 296 > 132 |
| IAA-Glu | 14,77 | 14,67 | 305 > 130 | d5: 311 > 134 |
| oxIAA-Glu | 11.7/ 12.2/ 14.5 | 11.75/ 12.2/ 14.5 | 321 > 146 | d2: 323 > 148 |
